# Does Physical Fitness Prior to Traumatic Brain Injury Affect Recovery Outcomes? A Scoping Review of Human and Animal Research

**DOI:** 10.1177/2689288X251376991

**Published:** 2025-09-08

**Authors:** Dean M. Cordingley, Izabella Marquez, Serena C.L. Buchwald, Frederick A. Zeiler

**Affiliations:** ^1^ Pan Am Clinic Foundation, Winnipeg, Manitoba, Canada.; ^2^ Applied Health Sciences Program, Faculty of Kinesiology and Recreation Management, University of Manitoba, Winnipeg, Canada.; ^3^ Department of Biosystems Engineering, Price Faculty of Engineering, University of Manitoba, Winnipeg, Canada.; ^4^ Department of Biomedical Engineering, Price Faculty of Engineering, University of Manitoba, Winnipeg, Canada.; ^5^ Section of Neurosurgery, Department of Surgery, Rady Faculty of Health Sciences, University of Manitoba, Winnipeg, Canada.; ^6^ Centre on Aging, University of Manitoba, Winnipeg, Canada.; ^7^ Division of Anaesthesia, Department of Medicine, Addenbrooke’s Hospital, University of Cambridge, Cambridge, UK.

**Keywords:** cognition, exercise, mild traumatic brain injury, physical activity, physiology

## Abstract

The objective of this study was to identify whether physical fitness influences outcomes following traumatic brain injury (TBI) in humans and animals, and to highlight any knowledge gaps in the current literature. A search of EMBASE, MEDLINE, SCOPUS, BIOSIS, and Cochrane Libraries was performed on December 4, 2023 (from database inception), and a systematic scoping review of the resulting literature was conducted. The Preferred Reporting Items for Systematic Reviews and Meta-Analyses extension for scoping reviews was used for reporting the results. An online systematic review management system was used to remove all duplicates and for subsequent article screening. Following the removal of duplicates, the search identified 6,818 articles for screening, of which 10 articles met the inclusion/exclusion criteria and were included. One study was conducted in humans, while the remaining nine utilized murine models. The available literature is limited and has investigated a large variety of outcomes that were not consistent across studies. Additionally, the pre-TBI exercise intervention or fitness assessment method varied between the studies. With the current literature, it is not possible to draw conclusions regarding the effects of physical fitness level and outcomes following a TBI.

## Introduction

Traumatic brain injuries (TBI) are highly prevalent and result in notable health, social, and economic burdens on the health care system. Of all TBIs, it is estimated that over 70–95% are mild TBI, which encompasses concussions.^1,2^ The primary mechanisms of mild TBI/concussions are age dependent, with the majority of those experienced by adolescents and young adults from sport and recreation activities,^3,4^ while older adults primarily experience concussions from falls.^5^ The typical recovery from a concussion is 10–14 days for adults, while recovery is longer for children, who typically take between 21 and 28 days.^6–10^ However, 10–30% of people who sustain a concussion develop persistent symptoms beyond the expected duration.^11–14^ Thus, it is imperative that modifying factors be studied to assist in establishing prophylactic and reparative therapeutic interventions to decrease long-term disability for patients as well as decrease the burden on health care systems.

Many studies have investigated the benefits of aerobic exercise as a treatment following mTBI and sport-related concussions (SRC).^15^ These studies have found that early exercise interventions following concussion lead to a lower risk of developing persisting concussion symptoms (PCS) and improve time to recovery,^16–18^ while aerobic exercise initiated in individuals with PCS can improve symptom severity.^19^ Pre-injury physical fitness has been identified as a modifiable factor that may be associated with more favorable outcomes in other brain injury models. Physical activity (PA) is a modifiable risk factor for stroke, with higher levels of regular PA reducing the risk of stroke.^20,21^ The relationships between PA and stroke risk may be due to the positive outcomes of PA, such as reduced blood pressure, diabetes prevention, and overall health.^22^ Recent literature suggests that pre-stroke PA may contribute to improved outcomes following stroke. It was suggested that PA may contribute to neuroprotection via the upregulation of neurotrophic and angiogenic factors.^23^ Further, pre-stroke PA may be associated with motor improvements after stroke.^24,25^ Although positive findings have been observed in stroke models, it is unclear if similar results would be yielded in TBI models. Therefore, the purpose of this study was to identify whether physical fitness influences outcomes following TBI in humans and animals, and to highlight any knowledge gaps in the current literature.

## Materials and Methods

The protocol for the scoping review was not registered. The scoping review was carried out following the Cochrane Handbook for Systematic Reviews and the scoping review extension for Preferred Reporting Items for Systematic Reviews and Meta-Analyses (PRISMA).^26^

### Research question

The research question asked was: “Does physical fitness level before a TBI change outcomes following the injury?”

Animal models were required to use a mechanically based injury model (i.e., controlled cortical impact, fluid percussion injury, and weight drop) instead of a pharmacologically induced TBI, and interpretation of injury severity was based on the author description.

Pre-TBI physical fitness was defined as any quantitative assessment of physical fitness level (cardiorespiratory and/or musculoskeletal systems) prior to the TBI-inducing event for humans, or a physical fitness intervention prior to the occurrence of the TBI, where a trained group was compared with an untrained group of participants.

Outcomes for this study included clinical outcomes (i.e., time to patient recovery), patient-reported outcomes (PROMs), as well as biomolecules and biomarkers. PROMs were considered outcomes that were reported by the patients relating to their condition, such as symptoms, quality of life, and functional and health status. For this study, biomolecules and biomarkers were defined as proteins, peptides, messenger or microRNA, which have been implicated as markers of inflammation, cell growth, cell death, or injury severity in the central nervous system. For a full list of included search terms, see Supplementary Data S1 for a sample of the search string.

### Search strategy

The study search strategy was developed by two researchers (D.M.C. and F.A.Z.) who have expertise on the topic, and the search was conducted on December 4, 2023. A search of EMBASE, MEDLINE, SCOPUS, BIOSIS, and Cochrane Libraries was conducted, and all identified literature was uploaded to an online systematic/scoping review management software (https://www.rayyan.ai). This software was used to remove duplicates and for screening the remaining articles by researchers (S.C.L.B., I.M., K.S., and D.M.C.). All included articles had their reference lists reviewed for any additional studies missed during the initial search. A sample of the search strategy (Supplementary Data S1) and the PRISMA checklist (Supplementary Data S2) are available.

### Eligibility criteria

#### Inclusion criteria

Articles were required to be published in a peer-reviewed journal and include either an exercise intervention or the assessment of physical fitness prior to the participant experiencing a TBI. Human and animal studies were both included, and participants of any age were allowed. Human studies required participants to have been diagnosed with a TBI only. Animal-based models were required to utilize only mechanically induced mild TBI (i.e., controlled cortical injury, fluid percussion injury, and weight drop).

#### Exclusion criteria

Articles that were determined to be a conference abstract/paper, case report, case series, or literature that was not original research were excluded. Studies that did not report a pre-TBI assessment of physical fitness or did not include an exercise intervention prior to the TBI were excluded. Additionally, studies that utilized a pharmacological model of brain injury and/or only reported on biomolecules or biomarkers not associated with the central nervous system were excluded. Additionally, studies that were non-English were excluded.

### Article selection process

All titles and abstracts were reviewed by three researchers (S.C.L.B., I.M., and K.S.) to determine for all articles that a full-text review would be performed. Three researchers (S.C.L.B., K.S., and/or D.M.C.) reviewed the full texts of all identified articles. An additional researcher (F.A.Z.) was included if there was a disagreement to assist in reaching consensus.

### Data charting process

Two templates (see Supplementary Tables S1A and S1B) were used for the extraction of study design, participant characteristics, methodologies, physical fitness assessment and/or exercise intervention protocol, all outcome measures, detailed PROMs and/or biomolecular and biomarker outcomes, study results specific to the influence of physical fitness prior to TBI on outcomes, and study conclusions.

### Data items

Extracted data included patient characteristics (including sample size, age, biological sex, and any additional relevant information related to the injury), study design, exercise intervention and/or physical fitness assessment, outcome measures, specific methods for PROMs and/or biomolecule and biomarker measures, study results, and general conclusions of the study.

### Bias assessment

A bias assessment was not performed for this study since the purpose was to identify whether physical fitness influences outcomes following TBI in humans and animals and to highlight any knowledge gaps in the current literature.

### Synthesis of results

No formal statistical analyses were performed. The data extracted from the studies were described to provide an overview and summary of the current literature on the topic.

## Results

### Search results and study characteristics

**FIG. 1.**
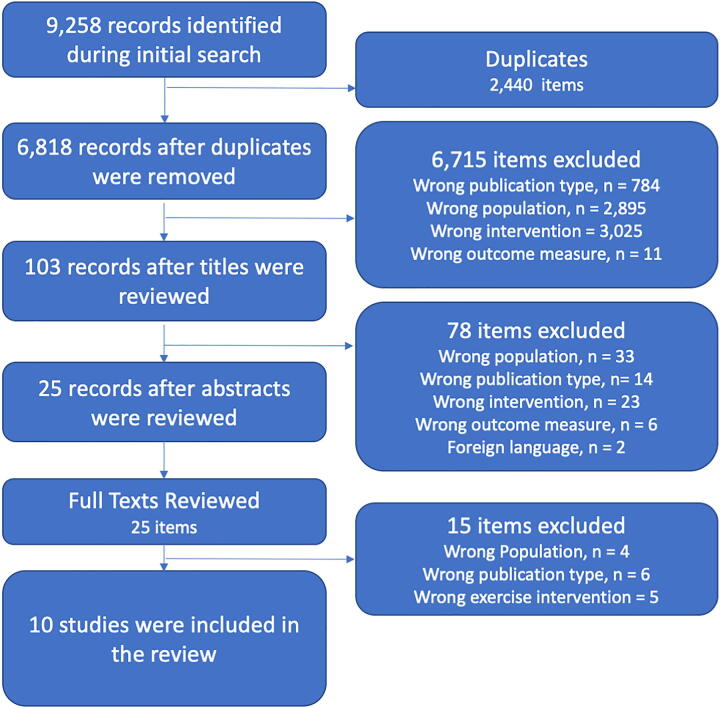
PRISMA flow diagram. PRISMA, Preferred Reporting Items for Systematic Reviews and Meta-Analyses.

There were 9,258 articles identified in the initial search of all included databases. A total of 2,440 articles were determined to be duplicates and deleted, leaving 6,818 articles for title screening. Title screening removed 6,715 articles, leaving 103 articles for abstract screening. Following review of the abstracts, 78 did not meet the inclusion/exclusion criteria and were removed. There were 25 articles that remained requiring full-text screening. Full-text screening resulted in 15 articles being excluded because they incorporated pre-injury and post-injury exercise as part of their intervention and it was not possible to distinguish between the effects of each (*n* = 5), the articles were not original peer-reviewed research (*n* = 6), or they included non-TBI (*n* = 4). There were 10 articles that were included following application of the search strategy and inclusion/exclusion criteria (see Fig. 1 for PRISMA flow diagram).

Of the included studies, one used a human design^27^ while nine used a murine model.^28–36^ There were five studies that investigated outcomes in concussion/mild TBI^27–31^ and five that utilized moderate/severe TBI models.^32–36^ The murine models consisted of five that used Wistar rats and four that used mice. The murine-based studies only used male rodents,^28–31^ while the lone human study utilized both male and female cadets.^27^ The one human study utilized a 1.5-mile run to predict maximal oxygen consumption ( $\dot{V}O$_2Max_) and determine participant physical fitness levels.^27^ The murine studies used a variety of exercise interventions to induce exercise adaptations prior to TBI induction. Two studies utilized a treadmill training program,^31,35^ four provided a running wheel for voluntary running,^29,32,34,36^ and three studies used a swimming intervention.^28,30,33^ A summary of study characteristics and outcome measures for individual studies is presented in Table 1. Detailed study characteristics and outcome measures of individual studies are presented in Supplementary Table S1A, and results and conclusions regarding the effects of physical fitness on post-TBI outcomes are presented in Supplementary Table S1B.

**Table 1.** Summary of Individual Study Characteristics and Outcome Measures

Reference	Sample	Injury model	Preconditioning	Tissue	Outcome in response to pre-injury fitness/preconditioning compared with no preconditioning
Lima et al.^28^	Wister rats	Mild FPI	5 days/week for 6 weeks, swimming	Brain tissue	↓ TBARS, carbonyl content ↑ Na^+^, K^+^-ATPase activity, Na^+^, K^+^-ATPase α1 subunit
Gu et al.^29^	C57BL/6J mice	Mild CCI	3 weeks, voluntary wheel running	Brain tissue	↓ latency time (MWM) ↑ percent in target quadrant (MWM), somata size, dendritic complex, apical branch points and basal branch points, NeuN, GAP43, COX-I, COX-II, COX-III, synapsin-I, SYP, BDNF, CcO activity, ATP ↔ GFAP
Da Silva Fiorin et al.^30^	Wistar rats	Mild FPI	5 days/week for 6 weeks, swimming	Brain tissue	↓ DCFH-DA, SEE ↑ object recognition, Na^+^, K^+^-ATPase activity, SOD activity, P-Nrf2, Hsp70 ↔ NS, glutamate uptake, EAAT1, EAAT2, SOD2, immunocontent of Hsp70
Chio et al.^31^	Wistar rats	Mild FPI	5 days/week for 3 weeks, treadmill	Systemic blood, brain tissue	↓ motor deficits, cerebral contusion, edema, neuronal loss, apoptosis, Ccl22, Cxcl10, IL-18, IL-16, Cd70, Faslg, Mif ↑ HSP70, Bmp6, IL-10, IL-22, IL-6 ↔ Ccl2, Ccl3, Ccl17, Ccl19, Cxcl19, Ppbp, IL-1rn, Il-7, Ltb, Tnfrsf11b, Bmp7, Gpi
Van Pelt et al.^27^	Cadets	SRC and non-SRC	Prior AFT and PFT	N/A	↔ days until asymptomatic, days until return to play
Taylor et al.^32^	C57/BL6 mice	Moderate CCI	6 weeks, voluntary wheel running	Brain tissue	↓ sensorimotor (foot faults during gridwalk task) and spatial learning memory deficits (RAWM). ↑VEGF-A mRNA and protein expression (cerebral cortex and hippocampus), EPO mRNA expression (cerebral cortex) ↔ EPO mRNA or protein expression (hippocampus), EPO protein expression (cerebral cortex), HO-1 mRNA expression
de Castro et al.^33^	Wistar rats	Severe FPI	5 days/week for 6 weeks, swimming	Trunk blood, liver tissue and brain tissue	↓ latency and errors (Barnes Maze), hippocampus cell loss, iNOS, COX2, TNF-α, IL-6, pJNK, DCFH-DA oxidation, SOD, ATP K_M_, blood insulin, blood glucose, fluorescein extravasation, MPO activity ↑ NS, glycogen content, LXR-α, ABCA1, pIRS, pAKT, free SH, GSH, CAT, MTT reduction, CS, Δψ, Na^+^, K^+^-ATPase activity, HOMA2%S ↔ ALT, AST
Zhao et al.^36^	C57BL/6NTac mice	Moderate CCI	4 weeks, voluntary running	Brain tissue	↓ latency (MWM), foot faults (beam walk test), immobile time (TS), lesion volume, activation of hypertrophic and bushy microglia, Bid, Puma, AIF-1, Cytochrome C, α-spectrin (150/145 kDa) ↑ time in target quadrant (Probe test), neuronal densities (CA1, CA2/3, DG, Cortex, and Thalamus) time with novel object, BDNF, CREB, HSPa1a, HSPa1b, HSP70 ↔ swimming speed, resting ramified microglia, α-spectrin (120 kDa), p53, Bak1, Akt, Noxa, Bim
Gan et al.^34^	C57BL/6J mice	Moderate FPI	6 weeks, voluntary wheel running	Brain tissue	↓ NSS, time to goal box (beam walking task), foot faults (grid walking task), time on grid (grid walking test) ↑ ankle angle (maximum take-off, swing, minimum stance) ↔ fall latency time (Rotarod task), swing velocity of (foot, ankle, and knee), stride length, foot vertical excursion, knee angle (maximum take-off, swing, minimum stance), TNF-α, IL-1β, IL-4, GFAP, LC3-II, lesion volume
Mota et al.^35^	Wistar rats	FPI	4 weeks, treadmill training	Brain tissue	↓ TNF-α, IL-1β, fluorescein, MPO ↑ NS, IL-10, Na^+^, K^+^-ATPase activity ↔ IL-6

FPI, fluid percussive injury; CCI, controlled cortical impact; SRC, sport-related concussion; AFT, aerobic fitness test; PFT, physical fitness test; TBARS, thiobarbituric acid-reactive substances; MWM, Morris water maze; COX, cytochrome c oxidase; GAP43, growth-associated protein 43; SYP, synaptophysin; BDNF, brain-derived neurotrophic factor; ATP, adenosine triphosphate; DCFH-DA, 2′,7′-dichlorofluorescein diacetate; SEE, spontaneous epileptiform events; SOD, superoxide dismutase; P-Nrf2, phosphorylated nuclear factor erythroid 2-related factor; Hsp, heat shock protein; NS, neuroscore; EAAT, excitatory amino acid transporter; Ccl, Chemokine (c–c motif) ligand; Cxcl, Chemokine (c–x–c motif) ligand; IL, interleukin; Cd70, CD70 antigen; Faslg, fas ligand; Mif, macrophage migration inhibitory factor; Bmp, bone morphogenetic protein; Ppbp, pro-platelet basic protein; Ltb, lymphotoxin beta; Tnfrsf, Tumor necrosis factor receptor superfamily; Gpi, glucose-6-phosphate isomerase; RAWM, radial arm water maze; VEGF-A, vascular endothelial growth factor A; EPO, erythropoietin; HO-1, heme oxygenase-1; iNOS, inducible nitric oxide synthase; TNF-α, tumor necrosis factor alpha; pJNK, phosphoralated c-Jun NH2-terminal kinase; ATP K_M_, Michaelis–Menten constant for ATP; MPO, myeloperoxidase; LXR-α, liver X receptor alpha; ABCA1, ATP-binding cassette transporter; pIRS, phosphorolated insulin receptor substrate; pAKT; free SH, nonprotein sulfhydryl; GSH, reduced glutathione; CAT, catalase; MTT, 3-(4,5-dimethylthiazol-2-yl)−2,5-diphenyltetrazolium bromide; CS, citrate synthase; Δψ, mitochondrial membrane potential; HOMA2%S, homeostasis model assessment; ALT, aminotransferase; AST, aspartate aminotransferase; PGC1α, proliferator-activated gamma coactivator 1-alpha; TS, tail suspension test; AIF-1, apoptosis inducing factor 1; DG, denate gyrus; CREB, cyclic adenosine monophosphate response element-binding protein; Bak1, BCL2-antagonist/killer 1; Akt, protein kinase B; NSS, neurological severity score; LC3-II, microtubule-associate proteins 1A/1B light chain 3; MPO, myeloperoxidase.

### Concussion/mild TBI

#### Human studies

The lone human study determined that physical fitness prior to a concussion does not influence time to recovery.^27^ The study enrolled 307 cadets (22.8% women) who sustained a concussion during the investigation period. Aerobic fitness test results from a 1.5-mile run and physical fitness test results (comprising pull-ups, standing long jump, push-ups, abdominal crunches, and a 600-yard run) from the most recent assessment prior to their concussion and the assessment earliest after their concussion were extracted from the cadets’ records. It was determined that pre-concussion aerobic fitness and physical fitness test results were not associated with the time to symptom resolution (*p* = 0.419 and *p* = 0.989, respectively) or the number of days until a subset were medically cleared to return to intercollegiate sport participation (*p* = 0.811 and *p* = 0.665, respectively).

#### Murine studies

The earliest study identified in the search by Lima et al. (2009) investigated the effects of 6 weeks of swim training on mild TBI-induced oxidative damage and neurochemical dysfunction in 90-day-old male Wistar rats.^28^ Mild TBI resulted in increased thiobarbituric acid-reactive substances (TBARS) and protein carbonyl content in the ipsilateral cortex 48 h after injury; however, swim training prevented increases (TBARS, *p* < 0.03; protein carbonyl, *p* < 0.007). Additionally, prior swim training also protected against mild TBI-induced decreases in Na^+^, K^+^-ATPase activity (*p* < 0.05) and α_1_ subunit of Na^+^, K^+^-ATPase immunocontent in the ipsilateral cerebral cortex (*p* < 0.05). The same group published another study that again utilized a 6-week swim training protocol to investigate some additional neurochemical markers as well as neuromotor impairments.^30^ Swim training prior to mild TBI resulted in decreased cell loss in the dentate gyrus hilus, the prevention of Na^+^, K^+^-ATPase activity decreases, partial protection of glutamate uptake decreases, maintained superoxide dismutase activity, 2′,7′-dichlorofluorescein diacetate oxidation, increased heat shock protein 70 (Hsp70) expression, increased brain-derived neurotrophic factor (BDNF) content, and increased phosphorylated nuclear factor erythroid 2-related factor content. However, there was no difference between the swim-trained and untrained groups in immunocontent or expression of excitatory amino acid transporter 1 (EAAT1) and EAAT2 following mild TBI. Additionally, a composite neuroscore was determined 24 h following fluid percussive injury (FPI) to evaluate neuromotor impairments. It was determined that mild TBI resulted in a decreased neuroscore compared with a group of sedentary rats that underwent sham surgery, but there was no difference when swim training preceded the mild TBI. However, there was no statistical difference in neuroscore between the mild TBI groups that received swim training and the group that did not. Taken together, these results suggest swim training prior to mild TBI may contribute to a partial protective effect against neuromotor impairments. Lastly, Da Silva Fiorin et al.^30^ investigated spontaneous epileptiform events (SEE) via electroencephalographic recordings and determined that swim training prior to mild TBI reduced the number of SEE events compared with the group that did not receive training prior to mild TBI.

Additional research groups have also investigated the effects of exercise preconditioning on neurochemical and neurocognitive outcomes following mild TBI in murine models. Gu et al.^29^ investigated 3 weeks of voluntary wheel running prior to a controlled cortical impact injury in male C57BL/6J mice who were 4–4.5 months of age. It was determined that voluntary running prior to mild TBI resulted in increased NeuN-immunoreactive positive neurons, increased protein content of growth-associated protein 43, cytochrome c oxidase (COX)-I, II, and III, synapsin-I, synaptophysin, and BDNF (all *p* < 0.05) compared with the mild TBI group that did not have running wheel access. Additionally, running wheel access prior to mild TBI assisted in maintaining mitochondrial function as determined by mediated decreases in Cytochrome C oxidase activity and adenosine triphosphate (ATP) amounts compared with those who did not have access to a running wheel and demonstrated greater decreases following mild TBI. However, there was no difference in glial fibrillary acidic protein-positive neurons (*p* < 0.05) between the group that voluntarily ran and the group that did not have access to a running wheel. In addition to the evaluation of neurochemical changes, it was found that voluntary running prior to mild TBI resulted in decreased escape latency during the Morris Water Maze compared with the group that did not perform voluntary running (*p* < 0.05).

The final study investigated the effects of exercise preconditioning on the inflammatory response after an FPI-induced TBI.^31^ It was found that 3 weeks of treadmill training prior to FPI inhibited mRNA expression of chemokine (c–x–c motif) ligand 19, IL-18, IL-16, CD70 antigen, macrophage migration inhibitory factor, and fas ligand and promoted the expression of bone morphogenetic protein 6, IL-10, IL-22, and IL-6. However, the 12 remaining biomarkers examined were not different between the exercise preconditioning and no-exercise preconditioning groups. Additionally, the rats that completed the treadmill preconditioning showed attenuated motor deficits, brain contusions, brain edema, and neuronal loss and apoptosis compared with the group that did not receive treadmill preconditioning prior to FPI. Interestingly, it is noted that the protective effects of exercise preconditioning may be mediated through HSP70.

### Moderate/severe TBI

There were five studies identified that examined exercise preconditioning before moderate/severe TBI in murine models.^32–36^ All identified studies evaluated some aspect of functional or behavioral outcome following TBI. Neurological severity scores,^33–35^ sensorimotor function,^32,34^ motor function,^34,36^ spatial learning,^32,36^ memory,^33,36^ locomotor activity,^36^ depressive-like behavior,^36^ and novel object recognition^36^ were all improved with exercise preconditioning compared with the TBI-only groups. Functional and behavioral improvements with exercise preconditioning may be due to the protective effects of exercise, since it may decrease lesion volume and increase neuronal densities in CA1, CA2/3, denate gyrus, cortex, and thalamus.^36^ However, some research suggests lesion volume may not be protected with exercise preconditioning.^34^ Additionally, exercise training prior to TBI may help maintain blood–brain barrier integrity post-TBI.^33^

Mechanistically, exercise training prior to TBI may increase neuroplasticity-related factors such as vascular endothelial growth factor,^32^ BDNF,^36^ and cAMP response element-binding protein.^36^ Additionally, exercise preconditioning may inhibit the pro-inflammatory response and promote an anti-inflammatory response post-TBI. The pro-inflammatory cytokines TNF-α,^33,35^ IL-6,^33^ and IL-1β^35^ may downregulate following TBI, while the anti-inflammatory cytokine IL-10^35^ could upregulate with exercise preconditioning. However, other studies have found no difference in pro- and anti-inflammatory cytokines in response to TBI with exercise preconditioning.^34,35^ The difference in findings may be due to multiple factors, including the type of rodents utilized, exercise preconditioning protocol, time post-TBI to sacrifice, and tissue analyzed.

Exercise may be protective against cell death and promote cell survival. TBI-induced upregulation and translocation of proapoptotic molecules can be inhibited with exercise preconditioning. Decreased levels of cytochrome-C, Bid, Puma, and AIF-1 have been observed with exercise preconditioning compared with a TBI-only group.^36^ Additionally, the expression of HSPa1a and HSPa1b (which encode HSP70) are upregulated following TBI with exercise preconditioning, while α-spectrin (which is a marker of cell death) is downregulated indicating the promotion of cell survival.^36^

Taken together, exercise preconditioning prior to TBI could enhance cognitive and motor function, possibly through the promotion of neuroplasticity-related factors and anti-inflammatory cytokines, while reducing pro-inflammatory cytokines and proapoptotic molecules. However, the current literature on the effects of exercise training prior to TBI is limited.

## Discussion

This study was conducted to identify whether physical fitness influences outcomes from a TBI in humans and animals, and to highlight any knowledge gaps in the current literature. This scoping review identified that only one research study in humans has investigated whether a person’s level of physical fitness influences their outcomes following a TBI.^27^ The lone study found no difference in time to symptom resolution or time to return to play based on physical fitness level following a concussion.^27^ The remaining nine studies were conducted using pre-clinical murine models and investigated numerous different biomolecules/biomarkers, neurocognitive, and behavioral outcomes.^28–31^ The biomolecule and biomarker outcomes ranged greatly between studies and included markers of inflammation, cell damage and repair, and mitochondrial function, and were collected from different tissues (i.e., brain tissue and systemic blood). Additionally, the preconditioning exercise protocols varied in duration, type, and intensity between the studies. Given the small number of studies and variations in outcomes and protocols, it is not possible to draw any clinically meaningful conclusions for human TBI populations. Further investigations on the effects exercise preconditioning has on concussion outcomes are needed.

Murine models that evaluated neurocognitive function following TBI identified potential benefits of exercise preconditioning. Rodents that underwent exercise preconditioning showed decreased latency time and improved targeting in the Morris water maze test^29^ and improved object recognition.^30^ Additionally, exercise preconditioning may assist in the recovery of motor and neurological deficits,^31–36^ but this finding is not consistent, as others have reported no benefit of exercise preconditioning on neurological deficits.^30^ A possible explanation for this discrepancy is the use of different tools to evaluate motor and neurological function.

Exercise preconditioning appears to mitigate morphological changes, neuron cell loss, and SEE as a result of TBI in rodent models.^29–31,33,36^ Mechanistically, these benefits may be from exercise-induced upregulation of endogenous anti-oxidation processes, resulting in a reduction of cellular oxidative damage.^28,30^ Completing voluntary running before a mild TBI improved mitochondrial function and oxidative phosphorylation-implicated enzymes, as well as increased the amount of ATP in mice brains.^29^ However, any observed benefits of exercise preconditioning do not appear to be due to changes in glutamate release or uptake.^30^

Concussion and mild TBI are known to result in a pro-inflammatory response that, if prolonged, may result in persistent post-concussion symptoms.^37^ PA is recognized to have anti-inflammatory effects that contribute to the prevention and treatment of diseases associated with chronic inflammation.^38^ One identified study investigated the effects of treadmill preconditioning prior to mild TBI on a panel of inflammatory markers and found some anti-inflammatory markers are upregulated, while pro-inflammatory markers are downregulated with preconditioning.^31^ These findings hold true for moderate and severe TBI models.^33,35^ Further research into the effects of exercise preconditioning on the concussion-induced inflammatory response in humans would be valuable to further understand this potential mechanism of action. Additionally, the moderate and severe TBI literature suggests that exercise preconditioning promotes cell survival by decreasing the expression of proapoptotic molecules and increasing the expression of genes associated with cell survival.^36^

### Limitations of the literature

The literature is greatly limited by the lack of human studies, as the translation of murine models of TBI to clinically meaningful outcomes in humans has had limited success.^39,40^ Additionally, there were only four murine studies that utilized varying preconditioning exercise interventions (i.e., voluntary running, treadmill running, and swimming), different durations of intervention (3 or 6 weeks), and different time points for the measurement of outcomes. Although the one human study included both males and females,^27^ all murine models included only males.^28–31^ Additionally, the lone human study did not utilize a controlled intervention, and due to all participants being cadets which requires a physical fitness standard, the range of fitness levels would be limited.^27^ The mix of intervention protocols, duration from injury to measurement of outcomes, and the lack of females does not provide enough information to draw generalized conclusions. Another limitation of the available literature is the limited crossover of outcome measures between studies, resulting in the inability to compare and contrast outcomes between the multitude of study designs and the few studies available.

### Limitations of this review

The authors’ ability to read and understand only English resulted in the exclusion of studies that were only published in another language. Additionally, many murine studies did not directly state the severity of TBI induced by their model. If it was not possible to explicitly determine that the model induced a moderate or severe TBI, and therefore the study was excluded, the study was included in the scoping review. Lastly, due to the small number of studies, the mix of interventions, and the varying outcome measures, it is not possible to draw conclusions from this review.

### Future directions

PA is known to have many beneficial effects on brain health, as well as the prevention and treatment of many diseases.^38,41^ Future research to determine if concussion and mild TBI outcomes may be altered based on physical fitness would expand on the current stroke-based TBI literature.^25,42^ Due to the development of new technologies that allow for more advanced and less invasive biomarker analysis and physiological monitoring, more human studies should be considered. This could include the use of serial live-time cerebral physiological measurements, advanced neuroimaging, and biomarkers of injury and neurotrophic factors. Additionally, the inclusion of PROMs, pre-, post-, and long-term, would help elucidate a more holistic understanding of the impact physical fitness prior to concussion has on a patient. Additionally, future research should include both males and females to determine variations in responses due to biological sex. Gender should also be considered in future research, as both sex and gender influence patient outcomes.^43,44^

## Conclusion

The available literature is too limited to conclude whether physical fitness influences outcomes from a concussion or mild TBI in humans and animals. The limited murine studies suggest exercise preconditioning may alter various biomarkers, as well as neurological and motor outcomes, but more research is needed. There was only one human study identified from the scoping review that only assessed patient-reported symptoms and the days to return to play.^27^ More human studies are needed that evaluate additional patient-reported and biological outcomes to better understand the associated implications and adaptations of physical fitness prior to concussion.

## Abbreviations Used

AIF: apoptosis inducing factor
ATP: adenosine triphosphate
BDNF: Brain-derived neurotrophic factor
cAMP: cyclic adenosine monophosphate
CcO: Cytochrome c oxidase
CNS: central nervous system
COX: Cytochrome c oxidase
EAAT: excitatory amino acid transporter
FPI: fluid percussive injury
GFAP: Glial Fibrillary Acidic Protein
Hsp: heat shock protein
IL: interleukin
PA: physical activity
PCS: persisting concussion symptoms
PRISMA: Preferred reporting items for systematic reviews and meta-analyses
PROMs: patient-reported outcomes
SEE: spontaneous epileptiform events
SRC: sport-related concussion
TBARS: thiobarbituric acid-reactive substances
TBI: traumatic brain injury
TNF: tumor necrosis factor
VO_2Max_: Maximal oxygen consumption
