# Supplementary material for: Does Physical Fitness Prior to Traumatic Brain Injury Affect Recovery Outcomes? A Scoping Review of Human and Animal Research
Source: Neurotrauma Rep. 2025 Sep 8;6(1):768–77. doi: 10.1177/2689288X251376991 (PMC12528846; doi:10.1177/2689288X251376991)
Supplement: Supplementary Data S1 [file 2689288x251376991_supplementary_data_s1.docx]

Supplemental File A. Sample Search Strategy

SCOPUS

(KEY(“Mild traumatic brain injury”) OR KEY (“Mild TBI”) OR KEY (“Brain injury”) OR KEY (“Head injury”) OR KEY (“Head trauma”) OR KEY (“Cerebral trauma”) OR KEY (“Brain trauma”) OR KEY (“Cerebral Injury”) OR KEY (“Concussion”) OR KEY (“Closed brain injury”) OR KEY (“Acute brain injury”) OR KEY (“Brain injuries”) OR KEY (“Cerebrovascular trauma”) OR KEY (“Mild concussion”) OR KEY (“Cerebral Concussion”) OR KEY (“Brain Injuries, Traumatic”))

AND

(KEY (“Exercise”) OR KEY (“Physical activit*”) OR KEY (“Aerobic exercise”) OR KEY (“Light intensity exercise”) OR KEY (“Moderate intensity exercise”) OR KEY (“Exercise training”) OR KEY (“Acute exercise”) OR KEY (“Leisure Activit*”) OR KEY (“Recreation”) OR Key (“Preventative Exercise*”) OR KEY (“Exercise Therapies”) OR KEY (“Physical Conditioning”) OR KEY (“Exercise Preconditinoing”) OR KEY (“Exercise Pre-conditioning”) OR KEY (“Physical Fitness*”) OR KEY (“Cardiorespiratory fitness*”) OR KEY (“Aerobic Fitness*”) OR KEY (“Vigorous Exercise”))

AND

(KEY (“Proteins”) OR KEY (“Peptides”) OR KEY (“myelin”) OR KEY (“neurofilament protein”) OR KEY (“ubiquitin”) OR KEY (“Glial fibrillary acidic protein”) OR KEY (“S100”) OR KEY (“micro RNA”) OR KEY (“brain derived neurotrophic factor”) OR KEY (“BDNF”) OR KEY (“Biomarker*”) OR KEY (“Marker, Biological”) OR KEY (“Biologic Marker”) OR KEY (“Marker, Biologic”) OR KEY (“Biological Markers”) OR KEY (“Biologic Markers”) OR KEY (“Markers, Biologic”) OR KEY (“Biomarker”) OR KEY (“Markers, Biological”) OR KEY (“Markers, Immunologic”) OR KEY (“Immune Markers”) OR KEY (“Markers, Immune”) OR KEY (“Marker, Immunologic”) OR KEY (“Immunologic Markers”) OR KEY (“Immune Marker”) OR KEY (“Marker, Immune”) OR KEY (“Immunologic Marker”) OR KEY (“Serum Markers”) OR KEY (“Markers, Serum”) OR KEY (“Marker, Serum”) OR KEY (“Serum Marker”) OR KEY (“Biochemical Marker”) OR KEY (“Markers, Biochemical”) OR KEY (“Marker, Biochemical”) OR KEY (“Biochemical Markers”) OR KEY (“Tau”) OR KEY (“Proteins, tau”) OR KEY (“tau Protein”) OR KEY (“Protein, tau”) OR KEY (“Chemokine*”) OR KEY (“Chemotactic Cytokine”) OR KEY (“Cytokine, Chemotactic”) OR KEY (“Intercrines”) OR KEY (“Chemotactic Cytokines”) OR KEY (“Cytokines, Chemotactic”) OR KEY (“Intercrine”) OR KEY (“Interferon”) OR KEY (“Interleukin*”) OR KEY (“Leukemia Inhibitory Factor”) OR KEY (“Inhibitory Factor, Leukemia”) OR KEY (“Differentiation-Stimulating Factor”) OR KEY (“Differentiation Stimulating Factor”) OR KEY (“D Factor”) OR KEY (“Myeloid Differentiation-Stimulating Factor”) OR KEY (“Differentiation-Stimulating Factor, Myeloid”) OR KEY (“Myeloid Differentiation Stimulating Factor”) OR KEY (“Cholinergic Differentiation Factor”) OR KEY (“Differentiation Factor, Cholinergic”) OR KEY (“Emfilermin”) OR KEY (“LIF”) OR KEY (“Lymphokines”) OR KEY (“Lymphocyte Mediators”) OR KEY (“Tumor necrosis factor”) OR KEY (“Necrosis Factors, Tumor”) OR KEY (“TNF Receptor Ligands”) OR KEY (“Receptor Ligands, TNF”) OR KEY (“Tumor Necrosis Factor Superfamily Ligands”) OR KEY (“Adipokines”) OR KEY (“Adipokine”) OR KEY (“Adipocytokine”) OR KEY (“Adipocytokines”) OR KEY (“Inflammation”) OR KEY (“Inflammations”) OR KEY (“Innate Inflammatory Response”) OR KEY (“Inflammatory Response, Innate”) OR KEY (“Innate Inflammatory Responses”) OR KEY (“Myelin basic protein”) OR KEY (“Post Concussion Syndrome*”) OR KEY (“Post-Concussion Syndrome*”) OR KEY (“Post-concussive Symptom*”) OR KEY (“Post concussive Symptom*”) OR KEY (“Post-concussion Symptom*”) OR KEY (“Post concussion Symptom*”) OR KEY (“Post-Concussion Symptom*”) OR KEY (“Chronic post-concussive syndrome*”) OR KEY (“Chronic post concussive syndrome*”) OR KEY (“Function Recoveries”) OR (“Function Recovery”) OR KEY (“Recovery of Function*”) OR KEY (“Patient-Relevant Outcome*”) OR KEY (“Patient Relevant Outcome*”) OR KEY (“Clinical Effectiveness”) OR KEY (“Treatment Effectiveness”) OR KEY (“Rehabilitation Outcome*”) OR KEY (“Treatment Outcome*”) OR KEY (“Treatment Efficacy”) OR KEY (“Clinical Efficacy”) OR KEY (“Quality of life”) OR KEY (“Life Quality”) OR KEY (“Health-related quality of life”) OR KEY (“Health Related Quality of Life”) OR KEY (“HRQOL”) OR KEY (“Cognition”) OR KEY (“Cognitive function*”) OR KEY (“Memory”) OR KEY (“Mental Recall”) OR KEY (“Retention”) OR KEY (“Spatial Memory”) OR KEY (“Short-term memory”) OR KEY (“Long-term memory”) OR KEY (“Return to Sport*”) OR KEY (“Return to Play”) OR KEY (“Return to Sporting Activit*”) OR KEY (“Resumption of Sporting Activit*”) OR KEY (“Sporting Activity Resumption*”) OR KEY (“Activities Resumption*”) OR KEY (“Return to Recreational Activit*”) OR KEY (“Recreational Activities Resumption*”) OR KEY (“Return to School*”) OR KEY (“Return to Work”))
