# Supplementary material for: Does Physical Fitness Prior to Traumatic Brain Injury Affect Recovery Outcomes? A Scoping Review of Human and Animal Research
Source: Neurotrauma Rep. 2025 Sep 8;6(1):768–77. doi: 10.1177/2689288X251376991 (PMC12528846; doi:10.1177/2689288X251376991)
Supplement: Supplementary Tables [file 2689288x251376991_supplementary_tables.docx]

Supplemental Table A. Study Characteristics

| **Reference** | **Study design** | **Sample** | **Study protocol** | **Aerobic exercise intervention** | **Outcome measures and methods of measurement** |
| --- | --- | --- | --- | --- | --- |
| Lima et al., (2009). | RCT | Male Wistar rats; weight = 270-320g; age = 90-day old; N = 48 | 6 groups were randomly assigned: (i) trained/naïve (did not undergo any operation); (ii) trained/sham craniotomy; (iii) trained/TBI; (iv) sedentary/sham craniotomy; (v) sedentary/TBI;(vi) sedentary/naïve. After the 6-wk preconditioning period, a lactate threshold swim test was performed.  TBI was induced by FPI, and 48 hrs later sacrifice occurred. | The rats engaged in a 6-wk training period consisting of 60-mins of swimming 5 d/wk. After a 1-wk adaptation period lasting the first week of training, the rats began swimming with a workload of 5% their body weight. | TBARS content was determined via lipid peroxides assay by thiobarbituric acid reaction.  Carbonyl protein content was determined via spectrophotometry  Na^+^, K^+^-ATPase activity was determined by the subtraction of ouabain-sensitive activity and overall activity without ouabain, both determined by colorimetric assay.  Immunodetection analysis of Na^+^,K^+^-ATPase α_1_ subunit and slot blot assay for quantification of subunit levels  Protein determination: proteins were quantified via the Bradford method |
| Gu et al. (2014). | Clinical Trial –it is not indicated if groups were randomly assigned or not. | Adult male C57BL/6J mice; n = 52, age = 4-4.5 mos; N = 52 | Four groups were assigned: (i) non-mTBI RW; (ii) mTBI RW; (iii) non-mTBI no-RW; (iv) mTBI no-RW.  mTBI was induced via CCI. All mice underwent MWM assessments 3 times/d during days 15 – 20 post-CCI. Sacrifice occurred on day 20 post-CCI following the last MWM test. | The running groups were placed in a habitat with voluntary access to a RW for three weeks prior to injury induction. The number of revolutions on the wheel was recorded through a receiver attached to the wheel. The non-exercise groups had their running wheel immobilized. | Golgi stain was used to assess cortical morphology. Somata diameter, dendritic length, and basal/apical branch points were quantified.  Immunohistochemistry (IHC) was used to quantify NeuN, GFAP, and GAP43.  Western blot was used to determine COX-I, COX-II, COX-III, synapsin-I, SYP, BDNF, and GAP43 protein content in the hippocampus.  Mitochondria protein concentration was determined by BCA protein assay.  Mitochondrial ATP content was analyzed using the ATP Colormetric/Luminescence Assay kit and CcO activity was determined with the Cytochrome c Oxidase kit.  The Morris Water Maze was utilized to assess special learning. |
| Da Silva Fiorin et al. (2016) | RCT | Adult male Wistar rats; N = 160; weight = 250-350g | 4 groups were randomly assigned: (i) sendentary/sham; (ii) sedentary/FPI; (iii) exercise/sham; (iv) exercise/FPI.  The exercise preconditioning was 6 wks in duration.  TBI was induced via FPI. Motor function was assessed 24 hours following FPI, object recognition was evaluated 2-wks following FPI, and sacrifice occurred immediately after. | Rats in the exercise groups engaged in a swim training protocol 60 mins/day, 5 times/week for a period of 6 weeks. After the adaptation period of one week, the rats began swimming with a workload of 5% their body weight. | Motor function was assessed via neuroscore composite test  Object recognition was determined as the cumulative time spent at exploring objects in the arena.  [H]-glutamate uptake was determined by liquid scintillation.  Na+, K+ -ATPase activity assessment: activity was determined by the subtraction of ouabain-sensitive activity and overall activity without ouabain, both determined by colorimetric assay  Protein expression of EAAT1, EAAT2, Nrf2, PNrf2, SOD2, Hsp70, BDNF, and b-actin were determined with Western Blot.  Estimation of ROS production: in the ipsilateral hippocampus was determined by fluorescence.  MnSOD activity: via color reaction  Total protein content in the ipsilateral hippocampus was measured colorimetrically.  SEE was determined via EEG implantation and analysis  The number of cells in the dentate hilus was determined via Giesma staining. |
| Chio et al., (2017). | RCT | Male Wistar rats (300-320g), N = 64 | 8 groups were randomly assigned: (i) no exercise sham siRNA-vector (EP^−^ + sham + siRNA-vector); (ii) no exercise sham siRNA-HSP70 (EP^−^ + sham + siRNA-HSP70); (iii) exercise sham siRNA-vector (EP^+^ + sham + siRNA-vector); (iv) exercise- sham siRNA-HSP70 (EP^+^+ sham + siRNA-HSP70); (v) no exercise TBI siRNA-vector (EP^−^ + TBI + siRNA-vector); (vi) no exercise TBI siRNA-HSP70 (EP^−^ + TBI + siRNA-HSP70); (vii) exercise TBI siRNA-vector (EP^+^ + TBI + siRNA-vector); and (viii) exercise TBI siRNA-HSP70 (EP^+^ + TBI + siRNA-HSP70).  1 day before and 1-3 days after FPI induced TBI, neurological motor function was evaluated.  Sacrifice occurred 3 days after FPI. | The preconditioned exercise group were trained with treadmill exercise 5 d/wk for 3 wks. 3 days of treadmill acclimation occurred utilizing electrical shocks as needed. Subsequent training did not utilize electric shock and was progressive in nature. Week 1 consisted of 30 min/d at 20 m/min, week 2 at 30 min/d at 30 m/min, and week 3 at 60 min/d at 30 m/min. | Acute neurological injury was measured using a modified neurological severity score  Cerebral contusion assay: Cerebral ischemia extent caused by TBI was measured using triphenyl tetrazolium chloride (TTC) staining procedures. Contusion was measured by negative TTC stains.  Evans Blue extravasations and brain water content Evans Blue injection.  Protein analysis and quantification by Western blot  Immunofluorescence staining: was used to measure the mean number of NeuN/DAPI/TUNEL triple-labeled cells,  Gene analysis: qPCR was utilized to quantify genes encoding 84 cytokines and chemokines. RT^2^ Profiler^TM^ PCR Array Rat Cytokines & Chemokines profiler array  Ingenuity pathway analysis: QIAGEN’s Ingenuity® Pathway Analysis to observe top five canonical pathways.  ELISA: IL-6 corticol levels  Chromatin immunoprecipitation assay: Used to evaluate NF-κB binding to the DNA elements in the IL-6 promoter regions with EpiTect ChIP qPCR assays kit. |
| Van Pelt et al., (2023). | Longitudinal study | Cadets who sustained Cx between 2015-2017, with pre and post Cx AFT and PFT scores, N=307 (female, n=70; male, n=237; mean age = 19.6 ±1.45 (female = 19.5 ±1.44, male = 19.7 ±1.45). | All participants who sustained a Cx between 2015-2017 had their data extracted from their medical records, including time to symptom resolution and time to return to play. Aerobic and physical fitness data of the concussed cadets were pulled for their AFT and PFT tests with the closest date prior to their Cx. | Retrospective data for pre- and post-injury aerobic fitness test were utilized. The AFT is a 1.5-mile run to predict VO_2max_. A score out of 500 is given, with 500 points being given to men with a run time under 7:45 and women with a run time under 8:55. No points are given to those who have a run time of over 14:00 for men and 16:06 for women. The PFT is a battery of tests composed of pull-ups, standing long jump, abdominal crunches, push-ups and 600-yard run which are each performed for 2-min with a 1-min recovery period between tests. | Days until symptom resolution was determined as the patient reporting minimal or no continuing Cx symptoms and scores are within limits of baseline testing.  Days until RTP was determined as the time from injury until physician approved return to unrestricted and full contact activities. |
| Taylor et al. (2015) | RCT | Male, 5-month old C57/BL6 mice (28-32 g), N=120 | Mice were randomized to either the exercise or sedentary condition as well as a TBI or no TBI condition. Following the 6-wk exercise training (or sedentary) period a moderate TBI was induced via CCI. 1-d prior to TBI and on days 1, 3 and 7 post-TBI, mice were assessed for sensorimotor function. Spatial learning memory was assessed 1-d prior to TBI and 7-d post-TBI. Mice were sacrificed on days 1, 3 and 7 post-TBI for biochemical and histological analysis. | The mice assigned to the exercise condition were provided free access to a running wheel for 6-wks prior to CCI. | Sensorimotor function was determined via the number of foot faults during the grid walk task.  Spatial learning memory was evaluated with the radial arm water maze.  VEGF-A, EPO, and HO-1 mRNA expression were determined for the hippocampus and right cerebral cortex with qRT-PCR.  VEGF-A and EPO protein expression were determined via immunohistochemistry. |
| De Castro et al. (2017) | RCT | Male, Wistar rats (250 – 350 g), total sample size is unclear. | Rats were randomly assigned to sedentary or exercise groups for 6-wks. Rats then underwent sham surgery or FPI 24-hrs post-last training session. 24-hrs following FPI or sham rats underwent neuromotor evaluation with some being immediately sacrificed while the rest underwent memory testing 24-hrs later and were then sacrificed | The rats undergoing exercise training completed 60-min swim training 5-d/wk for 6-wks with the addition of 5% body weight added. | Neuromotor function was determined via the neuroscore test.  Memory function was determined via Barnes Maze  Blood glucose was determined via Aviva Accu-check monitor (Roche Diagnostic Corp., Indianapolis, IN, USA).  Blood insulin was determined via ELISA.  Serum IL-6 and TNF-α were determined via ELISA.  Serum ALT and AST were determined via automatic analyser (Hitachi 7020 Hitachi, Tokyo, Japan).  Liver LXR-α, ABCA1, iNOS, COX-2 and β-actin were determined via RT-PCR.  Liver IL-6 and TNF-α were determined via ELISA.  Liver ROS production was estimated via DCFH-DA.  Liver free-SH was determined via Ellman’s Assay.  Liver total protein carbonyl content, SOD activity, CAT activity,MTT reduction and CS activity were determined via spectrophotometry.  Liver levels of GSH was determined via fluorometry.  Liver protein content of iNOS, pIRS, pJNK, pAkt and COX-2 were determined via Western blot.  Liver mitochondrial Δψ was extimated via changes in fluorescence of safranine-O.  Liver Na^+^,K^+^-ATPase activity was determined colorometrically.  Ipsilateral cortex MPO activity was determined via spectrophotometry.  Ipsilateral cortex IL-6 and TNF-α content was determined via ELISA.  Ipsilateral cortex Na^+^,K^+^-ATPase activity was determined via the potentiometric method.  Blood brain barrier integrity was determined via the amount of sodium fluorescein in the brain versus serum |
| Zhao et al. (2015) | RCT | Male, C57BL/6NTac mice (10-wks old, 20–25 g), N = 51 | Mice performed 4-wks of voluntary exercise or sedentary behaviour prior to CCI. A subset of mice were sacrificed 24-hrs post-TBI. The remaining mice were assessed for motor function (immediately before and days 1, 3, 7, 14 and 28 post-TBI). On days 14-17 post-TBI mice underwent platform training for the MWM prior to a standard probe test on day 18. The tail suspension test and open field test were performed on day 21 post-TBI, while the novel recognition test was performed on days 22 and 23 post-TBI. Sacrific occurred 28-days post-TBI. | The mice assigned to the exercise pre-conditioning group performed voluntary wheel running for 4-wks. | Motor function was assessed via beam walking task.  Spatial learning and memory were assessed via MWM.  Depression like behaviour was assessed via tail suspension test.  Locomotor activity was assessed via open field test.  Novel object recognition was assessed with two objects at opposite corners of an open field.  Lesion volume was determined via Cavalieri’s method.  Cell loss and microglia morphology were determined via optical fractionator method with stereology.  RNA was isolated with an miRNeasy Kit (Qiagen, Valencia, CA).  α-Spectrin, Bid, PUMA, HSP70, AIF-1 and cytochrome c were assessed via RT-PCR. |
| Gan et al. 2022 | RCT | Male C57BL/6J mice (5-wks old, 16 – 18 g); exercise group, n = 39; sedentary group, n = 36 | Mice were randomly assigned to the exercise condition or control condition and did not have access to a running wheel. Mice were further divided within their exercise or non-exercise groups to receive sham surgery, FPI induced moderate TBI, or TBI and Yisaipu. NSS was assessed prior to and 2-hrs, 1-day and 2-days post-TBI. On day 3 post-TBI mice were sacrificed for biomolecular analysis | Exercise preconditioning consisted of voluntary wheel running 6 d/wk. | Functional status was assessed via NSS.  Balance, grip strength and coordination were assessed via rotaroad and beam walking tests.  Motorsensory function was assessed via foot fault test.  Coordination and locomotion were assed via gait analysis.  Serum TNF-α, IL-1β, IL-4 and IL-6 were assessed via ELISA.  Protein expression of LC3-II, GFAP, GAPDH were determined via Western Blot.  Lesion volume was determined via Hematoxylin and eosin staining. |
| Mota et al. (2011) | RCT | Male, Wistar rats (90-days old, 220-260 g), total sample size is unclear | Rats underwent 3 days of treadmill familiarization, and were then randomized to 4-wk training program or sedentary conditions. Rats then underwent FPI, sham surgery, or no surgical procedure. 24-hrs post-FPI motor function was assessed and the animals were sacrificed. | Treadmill training occurred 7-d/wk for 4-wks. All training sessions started with a 5-min warm-up at 10-12 m/min. The training stimuli gradually increased from 30-min at 15 m/min, to 60-min at 24-26 m/min by the end of the training period. | Motor function was determined via the neuroscore test.  Cytokine concentrations were determined via ELISA  MPO and Na^+^,K^+^-ATPase activity determined colorimetrically via the Bradford method.  BBB permeability was determined via Fluorescein and anaylized on a fluorometer. |

RCT, Randomized Control Trial; TBI, traumatic brain injury; PID, Post Injury Day; Q-PCR, Quantitative real-time polymerase chain reaction; DNA, deoxyribose nucleic acid; RNA, Ribonucleic acid; Cx, Concussion; PFT, Physical Fitness Test; AFT, Aerobic Fitness Test; RTP, Return to play; HR, Heart Rate; BPM, Beats per Minute; VO2max, Maximal oxygen consumption; ATP, Adenosine Triphosphate; IMPACT, Immediate Post-Concussion Assessment and Cognitive Testing; TTC, Triphenyl tetrazolium chloride; MWM, Morris Water Maze; ELISA, Enzyme-Linked Immunosorbent Assay; RW, Running wheel; IHC, immunohistochemistry; COX, Cyclooxygenase; GAP, Growth associated protein; CcO, Cytochrome c Oxidase; SYP, Synaptophysin; BDNF, Brain-derived neurotrophic factor; GAPDH, glyceraldehyde-3-phosphate dehydrogenase; BCA, Bicinchoninic Acid; TBARS, Thiobarbituric acid reactive substances; SDS, Sodium Dodecyl Sulfate; HCl, Hydrochloric Acid; DNPH, 2,4-Dinitrophenylhydrazine; LT, Lactate threshold; FPI, Fluid percussion injury; ROS, Reactive oxygen species; DI, Discrimination index; EAAT, Excitatory amino acid transporter; Nrf2, Nuclear factor erythroid 2-related factor 2; SOD, Superoxide dismutase; HSP, Heat Shock Proteins; DCF, 7¢-dichlorofluorescein; MnSOD, manganese SOD; EEG, electroencephalography; NRCT, Non-randomized control trial; SEE, Spike and slow wave epileptiform event; FPI, fluid percussive injury; CCI, controlled cortical impact; SRC, sport related concussion; d, days; wk, weeks; AFT, aerobic fitness test; PFT, physical fitness test; TBARS, thiobarbituric acid reactive substances; MWM, Morris water maze; COX, cytochrome c oxidase; GAP43, growth associated protein 43; SYP, synaptophysin; BDNF, brain derived neurotrophic factor; ATP, adenosine triphosphate; DCFH-DA, 2’,7’-dichlorofluorescein diacetate; SEE, spontaneous epileptiform events; SOD, superoxide dismutase; P-Nrf2, phosphorylated nuclear factor erythroid 2-related factor; Hsp, heat shock protein; NS, neuroscore; EAAT, excitatory amino acid transporter; Ccl, Chemokine (c-c motif) ligand; CxCL, Chemokine (c-x-c motif) ligand; IL, interleukin; Cd70, CD70 antigen; Faslg, fasligand; Mif, macrophage migration inhibitory factor; Bmp, bone morphogenetic protein; Ppbp, pro-platelet basic protein; Ltb, lymphotoxin beta; Tnfrsf, Tumor necrosis factor receptor superfamily; Gpi, glucose-6-phosphate isomerase; RAWM, radial arm water maze; VEGF-A, vascular endothelial growth factor A; EPO, erythropoietin; HO-1, heme oxygenase-1; iNOS, inducible nitric oxide synthase; TNF-α, tumor necrosis factor alpha; pJNK, phosphoralated c-Jun NH2-terminal kinase; ATP K_M_, Michaelis–Menten constant for ATP; MPO, myeloperoxidase; LXR-α, liver X receptor alpha; ABCA1, ATP-binding cassette transporter; pIRS, phosphorolated insulin receptor substrate; pAKT; free SH, non-protein sulfhydryl; GSH, reduced glutathione; CAT, catalase; MTT, 3-(4,5-dimethylthiazol-2-yl)-2,5-diphenyltetrazolium bromide; CS, citrate synthase; Δψ, mitochondrial membrane potential; HOMA2%S, homeostasis model assessment; ALT, aminotransferase; AST, aspartate aminotransferase; PGC1α, proliferator-activated gamma coactivator 1-alpha; TS, tail suspension test;AIF-1, apoptosis inducing factor 1; DG, denate gyrus; CREB, cyclic adenosine monophosphate response element-binding protein; Bak1, BCL2-antagonist/killer 1; Akt, protein kinase B; NSS, neurological severity score; LC3-II, microtubule-associate proteins 1A/1B light chain 3; MPO, myeloperoxidase.

Supplemental Table B. Study results and conclusions

| **Reference** | **Brain Related Outcomes** | **Relevant Results** | **Conclusions** |
| --- | --- | --- | --- |
| Lima et al., (2009). | TBARS, NA+K+-ATPase activity levels, NA+K+-ATPase α_1_ subunit content, and protein content for protein carbonylation . | TBARS & carbonyl content increased following TBI, however exercise preconditioning resulted in no change from the shame preconditioned group.  Preconditioning protected against a decrease in Na+, K+ -ATPase activity in the ipsilateral cerebral cortex following TBI (P < 0.05).  TBI induced decreased immunocontent of Na+, K+ -ATPase α1 subunit is attenuated with exercise preconditioning (P < 0.05) | This study showed that exercise preconditioning could mitigate TBI induced oxidative damage that results in neuronal cell dysfunction. |
| Gu et al. (2014). | MWM escape latency, COX-I, COX-II, COX-III, synapsin-1, SYP, BDNF, and GAP43, ATP levels, CcO activity assay. | All injured mice showed significantly delayed escape latency on the MWM, however exercise preconditioning decreased escape latency compared to no exercise preconditioning (P < 0.05).  Exercise preconditioning counteracted the decrease in somata size (P < 0.05), dendritic complex (P < 0.05), apical branch points (P < 0.05) and basal branch points (P < 0.05) compared to no exercise preconditioning.  Exercise preconditioning resulted in a greater number of NeuN and GAP43 positive cells than the group that did not receive preconditioning (p < 0.05) but there was no difference in GFAP positive count.  Exercise pre-conditioning resulted in greater protein content of COX-I, COX-II, COX-III, Synapsin-I, SYP and BDNF compared to the no exercise group (all p < 0.05).  CcO activity and the amount of ATP are both increased following TBI with preconditioning compared to no preconditioning (p < 0.05) | These results suggest voluntary RW activity prior to TBI can reduce morphological changes to the brain and mitigate decreases in mitochondrial oxidative phosphorylation and counteract cognitive deficits compared to no RW access prior to TBI. |
| Da Silva Fiorin et al. (2016) | Neuroscore composite score, discrimination index for time spent investigating new objects, [H]-glutamate uptake, Na+ - K+ -ATPase activity, protein determination and expression of EAAT1, EAAT2, Nrf2, phosphorylated Nrf2 (PNrf2), SOD2, Hsp70, BDNF, and b-actin estimation of ROS production, MnSOD activity, and SEE. | -There were no differences in motor impairment between sedentary/TBI and exercise/TBI groups but the exercise group did not reduce neuroscore compared to sedentary/sham groups while the sedentary/TBI group did, indicating partial protection of physical exercise.  - TBI induced decreases in Na+ , K+ -ATPase were protected against by exercise preconditioning (P < 0.05).  - Glutamate uptake was decreased following TBI, however exercise preconditioning resulted in similar uptake to the sham groups (P > 0.05).  - Compared to TBI/sedentary rats, exercise preconditining increased the content of EAAT1 (P < 0.05) and EAAT2 (P < 0.05) in the hippocampus.  - SOD content was greater in the preconditioned group following TBI than the sedentary group (P < 0.05).  -TBI induced an increase in DCFH-DA oxidation, but this increase was prevented with exercise preconditioning (P < 0.05).  - Exercise preconditioning increased P-Nrf2/Nrf2 content and Hsp70 expression following FPI compared to the sedentary/TBI condition (P < 0.05)  -BDNF expression was increased following TBI in the preconditioned group (P < 0.05), but there was no difference in the sedentary/TBI condition compared to Sham (P > 0.05)  - Previous physical exercise improved object recognition and DI compared to the sedentary/TBI condition (P < 0.05)  - The exercise preconditioning resulted in fewer SEE compared to the sedentary/TBI condition (P < 0.05). | The results of this study indicates that an increase in Nrf2 expression protects against the early oxidative damage induced by TBI. As well, Hsp70 are involved in protecting the brain from TBI. Further, after TBI, a molecular system induced from pretraining can delay or prevent secondary cascades leading to neurobehavioral disability. Pre-training may protect the initial stages of cell injury induced by TBI by decreasing early oxidative damage and protects against cell loss and SEE with TBI. |
| Chio et al., (2017). | HSP70, Ccl2, Ccl3, Ccl17, Ccl19, Ccl22, Cxcl10, Cxcl19, Ppbp, Il-18, IL-16, IL-1rn, Il-7, Cd70, Faslg, Ltb, Tnfrsf 11b, Mif, Bmp6, Bmp7, gpi, IL-10, Il-22, IL-6, DAPI, NeuN, TUNEL, edema, neurological and motor functions | All motor deficits, brain contusions, brain edema, and neuronal loss and apoptosis at PID 3 were attenuated with EP+ compared to EP-.  6/9 proinflammatory genes were inhibited in EP^+^ + TBI rats but not in EP^-^ + TBI rats - Cxcl 10, IL-18, IL-16, Cd70, Mif, and Faslg were inhibited while Ppbp, Ltd, and Tnfrsf were not  Conversely, 4/14 anti-inflammatory genes after TBI were increased in EP^+^ +TBI rats but not EP^-^ +TBI rats - IL-10, IL-22, IL-6, and Bmp 6. | Exercise preconditioning could decrease gene expression of pro-inflammatory markers while increasing the expression of anti-inflammatory associated genes compared to no exercise pre-conditioning. |
| Van Pelt et al, 2023 | Time to symptom resolution, return to play recovery time. | Preinjury physical fitness was not associated with time to symptom restoration to baseline or time to return to play (p>0.05) | Pre-concussion physical fitness levels did not change the time to asymptomatic or return to play. |
| Taylor et al. (2015) | Sensorimotor function,  spatial learning memory, VEGF-A, EPO, and HO-1. | Pre-TBI physical training resulted in better sensorimotor function (p < 0.01) and spatial learning memory (p < 0.05) compared to the no-exercise group after TBI.  VEGF-A mRNA (p < 0.01) and protein (p < 0.05) expression was greater in the cerebral cortex and hippocampus (mRNA, p < 0.01; protein, p < 0.05) 1-d post TBI with prior physical training compared to no physical training. EPO mRNA expression was higher in the cerebral cortex (p < 0.01) but not the hippocampus with physical training compared to no physical training. There was no difference between the physical training and no-physical training groups in EPO protein expression or HO-1 mRNA expression at any time-points. | Exercise preconditioning results in increased expression of neuroprotective genes, and better sensorimotor and spatial learning memory outcomes following TBI. |
| De Castro et al. (2017) | Neuromotor function, memory, blood glucose and insulin, IL-6, TNF-α, MPO activity, Na^+^,K^+^-ATPase activity, BBB integrity, ALT, AST, LXR-α, ABCA1, iNOS, COX-2, DCFH-DA, free-SH was determined via Ellman’s Assay, protein carbonyl content, SOD activity, CAT activity, MTT reduction, CS activity,  GSH, pIRS, pJNK, pAkt, and  Δψ. | Compared to the sedentary rats, those who underwent exercise preconditioning demonstrated improved memory (p < 0.05) and neuromotor function (p < 0.05). Exercise preconditioning resulted in decreased hippocampus cell loss (p < 0.05), decreased iNOS mRNA expression and protein content (p < 0.05) and decreased COX2 mRNA and protein expression (p < 0.05). Additionally, hepatic, serum and cerebral levels of TNF-α and IL-6 were all decreased following TBI with exercise pre-conditioning compared to the sedentary group (all p < 0.05). pJNK, DCFH-DA oxidation, SOD, ATP K_M_, blood insulin, blood glucose, fluorescein extravasation and MPO activity were all decreased (all p < 0.05) following TBI with prior exercise training compared to the sedentary group. Hepatic glycogen content, LXR-α expression and ABCA1 expression were all elevated following TBI with prior exercise training compared to the sedentary condition (all p < 0.05). pIRS, pAKT, free SH, GSH, CAT, MTT reduction, CS, Δψ, and HOMA2%S levels were all higher (all p < 0.05) in the exercise training group compared to the sedentary condition following TBI. Prior exercise training inhibited TBI induced decreases in both hepatic and cerebral Na^+^,K^+^-ATPase activity compared to the sedentary condition (both p < 0.05).  However, exercise preconditioning did not result in differences in ALT and AST concentrations compared to the sedentary group. | Previous exercise training may alter the inflammatory and oxidative response to TBI and protect against acute hyperglycemia post-TBI. |
| Zhao et al. (2015) | Motor function, memory, spatial learning, depressive like behaviour, locomotor activity, novel object recognition, microglia morphology, Bid, Puma, AIF-1, Cytochrome C, α-Spectrin,  neuronal densities (CA1, CA2/3, DG, Cortex, and Thalamus), BDNF, CREB, HSPa1a, HSPa1b, HSP70, p53, Bak1, Akt, Noxa and Bim. | Exercise preconditioning resulted in decreased latency time on MWM (p < 0.05), increased time in target quadrent of the Probe test (p < 0.05), and increased time with the novel object (p < 0.05) compared to group that did not receive exercise preconditioning suggesting improved cognitive function.  Foot faults during beam walking (p < 0.05 – 0.001, dependent on day post-injury) and immobility time during tail suspension (p < 0.001) were decreased with exercise preconditioning.  Exercise preconditioning decreased lesion volume (p < 0.01), decreased neural loss in CA1 (p < 0.05), CA2/3 (p < 0.05), DG (p < 0.001), cortex (p < 0.05) and thalamus (p < 0.001) regions, and reduced hypertrophic ( p < 0.001) and bushy (p < 0.001) microglia compared to the sedentary group.  BDNF (p < 0.001), CREB (p < 0.001), HSPa1a (p < 0.001), HSPa1b (p < 0.001), and HSP70 (p < 0.001) were upregulated with exercise preconditioning compared to the sedentary group.  Exercise preconditioning decreased the expression of Bid (p < 0.001), Puma (p < 0.001), decreased AIF-1 (p < 0.05) and Cytochrome C translocation (p < 0.001), and decreased cleavage of α-Spectrin (150/145 kDa) (p < 0.001) | Exercise preconditioning can improve outcomes post-TBI by activating anti-inflammatory and anti-apoptotic pathways. |
| Gan et al., (2022) | NSS, time to goal box (beam walking task), foot faults (grid walking task), time on grid (grid walking test),fall latency time (Rotarod task), gait kinematics, TNF- α, IL-1β, IL-4, GFAP, LC3-II, lesion volume | Exercise preconditioning resulted in a decreased NSS 2-hrs post-TBI (p < 0.001), time to goal box (beam walking task; p < 0.001), foot faults (grid walking task; p < 0.05), and time on grid (grid walking test; p < 0.001) compared to the TBI only group.  Gait kinematics for ankle angle during maximum take-off (p < 0.001), swing (p < 0.01), and minimum stance (p < 0.01) were all increased in the exercise preconditioning group compared to TBI only.  Exercise preconditioning did not result in any differences in fall latency time, swing velocity of (foot, ankle and knee), stride length, foot vertical excursion, knee angle (maximum take-off, swing, minimum stance), TNF- α, IL-1β, IL-4, GFAP, LC3-II or lesion volume compared to the TBI only group. | Exercise preconditioning improves post-TBI functional status and motor sensory function. |
| Mota et al. (2011) | Motor function, TNF- α, IL-1β, fluorescein, MPO, IL-10, Na^+^,K^+^-ATPase activity, IL-6 | Physical training prior to TBI is protective for NS evaluation of motor function. Prior physical training also protected against decreases in IL-10 (p < 0.05), and increases in TNF-α (p < 0.002) and IL-1β (p < 0.05), but did not alter IL-6 content (p > 0.05). The physical training decreased fluorescein extravasation (p < 0.05) and MPO activity (p < 0.001) induced by TBI, and increased Na^+^,K^+^-ATPase activity (p < 0.05). | Physical training prior to TBI may decrease the cerebral inflammatory response, preserve BBB integrity and preserve motor function. |

AFT, Aerobic Fitness Test; PFT, Physical Fitness Test; IMPACT, Immediate Post-Concussion Assessment and Cognitive Testing; PTA, Post traumatic amnesia; mRNA, messenger Ribose nucleic acid; PID, Post-Injury Day; siRNA, small interfering Ribose nucleic acid; IL-6, Interleukin-6; EP, exercise preconditioning; HSP70, heat shock protein 70; NF-kB, nuclear factor kabba-B; TBI, traumatic brain injury; MWM, Morris Water Maze; IHC, Immunohistochemistry; OD, Optical Density; COX, Cyclooxygenase; GAP, Growth associated protein; CcO, Cytochrome c Oxidase; SYP, Synaptophysin; BDNF, Brain-derived neurotrophic factor; GAPDH, glyceraldehyde-3-phosphate dehydrogenase; BCA, Bicinchoninic Acid; IP, Immunoreactive positive; GFAP, Glial fibrillary acidic protein; NeuN, Neuronal nuclei; TBARS, Thiobarbituric acid reactive substances; ATP, Adenosine triphosphate; ROS, Reactive oxygen species; DCFH-DA, 2′,7′-dichlorofluorescein diacetate; FPI, Fluid percussion injury; ROS, Reactive oxygen species; DI, Discrimination index; EAAT, Excitatory amino acid transporter; Nrf2, Nuclear factor erythroid 2-related factor 2; SOD, Superoxide dismutase; HSP, Heat Shock Proteins; DCF, 7¢-dichlorofluorescein; MnSOD, manganese SOD; EEG, electroencephalography; SEE, Spike and slow wave epileptiform event; RNS, Reactive Nitrogen Species; RCT, Randomized Control Trial; TBI, traumatic brain injury; PID, Post Injury Day; Q-PCR, Quantitative real-time polymerase chain reaction; DNA, deoxyribose nucleic acid; RNA, Ribonucleic acid; Cx, Concussion; PFT, Physical Fitness Test; AFT, Aerobic Fitness Test; RTP, Return to play; HR, Heart Rate; BPM, Beats per Minute; VO2max, Maximal oxygen consumption; ATP, Adenosine Triphosphate; IMPACT, Immediate Post-Concussion Assessment and Cognitive Testing; TTC, Triphenyl tetrazolium chloride; MWM, Morris Water Maze; ELISA, Enzyme-Linked Immunosorbent Assay; RW, Running wheel; IHC, immunohistochemistry; COX, Cyclooxygenase; GAP, Growth associated protein; CcO, Cytochrome c Oxidase; SYP, Synaptophysin; BDNF, Brain-derived neurotrophic factor; GAPDH, glyceraldehyde-3-phosphate dehydrogenase; BCA, Bicinchoninic Acid; TBARS, Thiobarbituric acid reactive substances; SDS, Sodium Dodecyl Sulfate; HCl, Hydrochloric Acid; DNPH, 2,4-Dinitrophenylhydrazine; LT, Lactate threshold; FPI, Fluid percussion injury; ROS, Reactive oxygen species; DI, Discrimination index; EAAT, Excitatory amino acid transporter; Nrf2, Nuclear factor erythroid 2-related factor 2; SOD, Superoxide dismutase; HSP, Heat Shock Proteins; DCF, 7¢-dichlorofluorescein; MnSOD, manganese SOD; EEG, electroencephalography; NRCT, Non-randomized control trial; SEE, Spike and slow wave epileptiform event; FPI, fluid percussive injury; CCI, controlled cortical impact; SRC, sport related concussion; d, days; wk, weeks; AFT, aerobic fitness test; PFT, physical fitness test; TBARS, thiobarbituric acid reactive substances; MWM, Morris water maze; COX, cytochrome c oxidase; GAP43, growth associated protein 43; SYP, synaptophysin; BDNF, brain derived neurotrophic factor; ATP, adenosine triphosphate; DCFH-DA, 2’,7’-dichlorofluorescein diacetate; SEE, spontaneous epileptiform events; SOD, superoxide dismutase; P-Nrf2, phosphorylated nuclear factor erythroid 2-related factor; Hsp, heat shock protein; NS, neuroscore; EAAT, excitatory amino acid transporter; Ccl, Chemokine (c-c motif) ligand; CxCL, Chemokine (c-x-c motif) ligand; IL, interleukin; Cd70, CD70 antigen; Faslg, fasligand; Mif, macrophage migration inhibitory factor; Bmp, bone morphogenetic protein; Ppbp, pro-platelet basic protein; Ltb, lymphotoxin beta; Tnfrsf, Tumor necrosis factor receptor superfamily; Gpi, glucose-6-phosphate isomerase; RAWM, radial arm water maze; VEGF-A, vascular endothelial growth factor A; EPO, erythropoietin; HO-1, heme oxygenase-1; iNOS, inducible nitric oxide synthase; TNF-α, tumor necrosis factor alpha; pJNK, phosphoralated c-Jun NH2-terminal kinase; ATP K_M_, Michaelis–Menten constant for ATP; MPO, myeloperoxidase; LXR-α, liver X receptor alpha; ABCA1, ATP-binding cassette transporter; pIRS, phosphorolated insulin receptor substrate; pAKT; free SH, non-protein sulfhydryl; GSH, reduced glutathione; CAT, catalase; MTT, 3-(4,5-dimethylthiazol-2-yl)-2,5-diphenyltetrazolium bromide; CS, citrate synthase; Δψ, mitochondrial membrane potential; HOMA2%S, homeostasis model assessment; ALT, aminotransferase; AST, aspartate aminotransferase; PGC1α, proliferator-activated gamma coactivator 1-alpha; TS, tail suspension test;AIF-1, apoptosis inducing factor 1; DG, denate gyrus; CREB, cyclic adenosine monophosphate response element-binding protein; Bak1, BCL2-antagonist/killer 1; Akt, protein kinase B; NSS, neurological severity score; LC3-II, microtubule-associate proteins 1A/1B light chain 3; MPO, myeloperoxidase.
